# Antioxidant Activity in *Rheum emodi* Wall (Himalayan Rhubarb)

**DOI:** 10.3390/molecules26092555

**Published:** 2021-04-27

**Authors:** Sang Koo Park, Yoon Kyung Lee

**Affiliations:** 1 Food Safety Management Division, Seoul Regional Korea Food and Drug Administration, Seoul 07978, Korea; 2 Department of Agriculture, Forestry and Bioresources, Plant Genomics and Breeding Institute, Research Institute for Agriculture and Life Sciences, Seoul National University, Seoul 08826, Korea; nknglee2403@snu.ac.kr

**Keywords:** *Rheum emodi* wall, himalayan rhubarb, polyphenol content, antioxidant activity, DPPH, SOD, ABTS

## Abstract

Using natural products as antioxidant agents has been beneficial to replace synthetic products. Efforts have been made to profile the antioxidant capacities of natural resources, such as medicinal plants. The polyphenol content of Himalayan rhubarb, *Rheum emodi* wall, was measured and the antioxidant activity was determined using DPPH and ABTS^+^ assay, and the oxidative stress was assessed using SOD enzymatic assay. Five different solvent fractions, *n*-hexane, *n*-butanol, ethyl acetate, dichloromethane, and water, were used for screening the antioxidant capacity in effort to determine the optimum extraction solvent. The total phenolic contents for *R. emodi* fractions ranged from 27.76 to 209.21 mg of gallic acid equivalents (GAE)/g of dry weight. DPPH and ABTS^+^ assay results are presented into IC_50_ values, ranged from 21.52 to 2448.79 μg/mL and 90.25 to 1718.05 μg/mL, respectively. The ethyl acetate fraction had the highest antioxidant activity among other fractions. Also, *n*-butanol and water fractions showed significantly lower IC_50_ values than the positive control in DPPH radical scavenging activity. The IC_50_ values of SOD assay of fractions ranged from 2.31 to 64.78 μg/mL. A similar result was observed with ethyl acetate fraction showing the highest SOD radical scavenging activity. The study suggests that the ethyl acetate fraction of *R. emodi* possess the strongest antioxidant activity, thus the most efficient in extracting antioxidant contents. Moreover, a highly significant correlation was shown between total polyphenol content and antioxidant activity screening assays. The compounds related to the antioxidant activity of *R. emodi* were identified to myricitrin, myricetin 3-galloyl rhamnoside, and myricetin, which have not been reported in studies about *R. emodi* before.

## 1. Introduction

*Rheum emodi* wall. ex Meissn. (Polygonceae) is a leafy perennial herb distributed in altitudes ranging from 2800 to 3800 m in the temperate and subtropical regions of Himalayas, from Kashmir to Sikkim in India. The rhubarbs, rhizomes of *Rheum* species are used in remedies of blood stagnation syndrome, which includes diabetes, atherosclerosis, ischemia, and inflammation in Japanese and Chinese traditional medicine. Several antioxidants and xanthine oxidase inhibitors have been isolated from methanolic extract of rhubarbs, and these properties have been linked to the multiple beneficial effects in these disorders [1,2]. *Rheum emodi* wall is known to contain several secondary metabolites, of which anthraquinones (such as emodin and rhein) are considered as the active ingredient [3]. The methanolic and aqueous extracts of *Rheum emodi* rhizome is known to contain high number of phenolic compounds and show Fe^3+^ reducing antioxidant properties [4]. It is known that fluid–fluid partition using solvents with different polarity is an efficient purification method for plant polyphenols [5]. However, phenolic compounds in different plant materials have their own unique polarities and chemical characteristics [6], so identifying effective solvent for purification of polyphenols for *R. emodi* is in demand.

According to Rolta et al., the total polyphenol contents and total flavonoids contents of sub-fractions of *Rheum emodi* rhizome were 417.94 mg g^−1^ GAE and 187.40 mg g^−1^ RE, respectively. The chloroform sub-fraction also showed antimicrobial activity. The LC/MS analysis identified phytocompounds, like emodin D4, rhein13c6, chrysophanol dimethyl ether, and resveratrol in the study [7,8]. The authors suggest these compounds can act as bioavailability enhancers of antibacterial and antifungal antibiotics. Singh and Chaturvedi conducted phytochemical profile including phenolic content, flavonoid content, and antioxidant activity of methanolic extracts of different *R. emodi* plant parts. The study revealed presence of phytochemicals like phenols, flavonoids, alkaloids, carbohydrates, proteins, anthraquinones, quinones, and glycosides, thus suggesting other plant parts of the rhizome could be assets in utilizing the materials to pharmaceutical industries [9]. However, this study failed to present the composition of beneficial compounds.

Free radicals produced by radiation, chemical reactions, and several redox reactions of various compounds may contribute to protein oxidation, DNA damage, and lipid peroxidation in living tissues and cells [10,11]. Most modern diseases are in progress from acute to chronic metabolic diseases. These are thought to be involved in free radicals, reactive oxygen species, and oxidative stress. Free radicals occur in everyday life, and most are removed by enzymes, like superoxide dismutase (SOD) [12,13,14,15]. However, excessive reactive oxygen species are suggested to be strongly associated with cellular aging and certain metabolic diseases [16,17].

Synthetic antioxidants, frequently used to preserve foods, are butylated hydroxyanisole (BHA), butylated hydroxytoluene (BHT), and tertbutyl hydroquinone (TBHQ). Reports on the toxicity of BHA and BHT, high manufacturing costs, and low consumer acceptance, with regard to the safety of food additives, have garnered a need for identifying an alternative natural and probably safer source of food antioxidant [18,19]. Therefore, it has been concentrated on the use of therapeutic natural products to screen for more effective antioxidant agents, as well as to elucidate the mechanisms of free radical prevention. Oriental medicinal plants have been of particular interest as a source of new agents, due to their variety in species and use as traditional medicines for many decades in Asia [20,21,22].

In this study, the antioxidant activity of different solvent fractions of Rhizome of *R. emodi* wall was investigated using several methods by comparing DPPH, SOD activity, ABTS^+^ radical scavenging activity, and total phenolic contents. The fractions were measured and evaluated to determine their correlation with antioxidant activity. Also, the antioxidative compounds of *Rheum emodi* wall extract was isolated and identified by using liquid chromatography with diode array detection and electrospray ionization/mass spectrometry (LC-DAD-ESI/MS).

## 2. Results

### 2.1. Total Phenolic Contents of R. emodi Fractions

To evaluate the antioxidant activity of *R. emodi*, five different solvents were used in extraction. The total phenolic content was measured using the Folin–Ciocalteu method and determined by a regression equation of calibration curve (Y = 0.009X + 0.0428, R^2^ = 1), expressed as gallic acid equivalents (GAE). As shown in Table 1, the value varied from 27.76 ± 0.50 mg GAE/g to 209.21 ± 5.81 mg GAE/g.

Among the fractions, ethyl acetate fraction showed the highest phenolic content, 209.21 mg GAE/g, followed by water (139.01 mg GAE/g), *n*-butanol (133.49 mg GAE/g), dichloromethane (66.27 mg GAE/g), and *n*-hexane (27.76 mg GAE/g).

### 2.2. Antioxidant Activity Analysis

The antioxidant properties of *R. emodi* fractions were screened using several methods. DPPH, ABTS^+^ radical scavenging activity, and SOD activity of the fractions and positive control, l-ascorbic acid, was measured into IC_50_ values (Table 2).

DPPH radical scavenging activity of the fractions ranged from 21.52 to 2448.79 μg/mL, while the IC_50_ value of positive control was 70.33 μg/mL. A lower IC_50_ value implies a larger scavenging activity. The IC_50_ values of the ethyl acetate (21.52 μg/mL), *n*-butanol (38.67 μg/mL), and water (43.04 μg/mL) fractions were significantly lower than that of l-ascorbic acid.

ABTS^+^ radical scavenging activity of fractions varied from IC_50_ value of 90.25 to 1718.05 μg/mL, whereas that of positive control was 111.06 μg/mL. The ethyl acetate fraction was found to have the highest ABTS^+^ radical scavenging activity among the other fractions, and its IC_50_ value was significantly lower than that of the positive control.

SOD radical scavenging activity of the fractions ranged from IC_50_ value of 2.31 to 43.87 μg/mL, and that of positive control was 64.78 μg/mL. The ethyl acetate fraction had the highest SOD radical scavenging activity, with an IC_50_ value of 2.31 μg/mL, followed by *n*-butanol (24.36 μg/mL), water (32.25 μg/mL), and dichloromethane (43.87 μg/mL). Moreover, IC_50_ values of the ethyl acetate, *n*-butanol, water, and dichloromethane fractions were significantly lower than that of l-ascorbic acid. On the other hand, the SOD radical scavenging activity was not detected in *n*-hexane fraction.

### 2.3. Correlation of Total Phenolic Content and Antioxidant Activity

The correlation of total polyphenol content in *R. emodi* fractions and the antioxidant activities have been analyzed (Table 3).

A highly significant correlation was observed between total phenolic contents and the antioxidant activity determined by SOD radical (R^2^ = −0.928, *p* < 0.01), DPPH radical (R^2^ = −0.765, *p* < 0.01), and ABTS^+^ radical (R^2^ = −0.740, *p* < 0.01).

### 2.4. Identification of the Antioxidant Compounds

**Figure 1:**
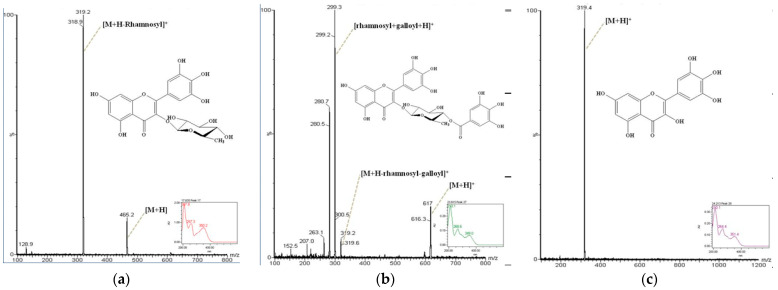
HPLC profiles, UV spectrum, and chemical structures of identified antioxidant compounds. (**a**) Myricitrin; (**b**) myricetin 3-galloyl rhamnoside; (**c**) myricetin.

The ethyl acetate fraction showed the highest polyphenols and antioxidant activity, thus selected as an extraction solvent for further analysis on the identification of the antioxidant compounds by HPLC with gradient elution. To identify the compounds, isolation was carried out by preparative HPLC separation using ethyl acetate fraction of *Rheum emodi* wall. As shown in Figure 1, several eluted phytochemicals in the ethyl acetate fraction were detected and showed positive peaks on the UV detector (254 nm and 350 nm). The compounds were identified as myricitrin (sym. myricetin 3-rhamnoside), myricetin 3-galloyl rhamnoside, and myricetin, in comparison with data from the literature, and each of the structures were drawn. The molecular ion peaks from ZQ MS of each compound were *m*/*z* 319, *m*/*z* 299, and *m*/*z* 319, respectively. The identified molecular weight of myricitrin, myricetin 3-galloyl rhamnoside, and myricetin was 464 g/mol, 616 g/mol, and 318 g/mol, respectively.

## 3. Discussion

Phenolics or polyphenols are secondary plant metabolites that are ubiquitously present in plants and their products [23]. Phenolic content in plants is known to be related to their antioxidant activities, explained by their probable redox properties, which allow them to act as reducing agents, hydrogen donors, and singlet oxygen quenchers [24]. The recovery of the phenolic contents from natural antioxidant plant sources could improve their added value. Thus, it is important to optimize the extraction process to obtain the maximum yield of these substances. In this study, a variation of the phenolic composition was observed based on different extraction solvent. Specifically, ethyl acetate fraction showed the highest phenolic content among the fractions. Akouwah et al. [25] suggested that the stability of extracts depends on the extraction solvent used for the removal of phenolic compounds, with respect to their antioxidant concentration and activities. The highest total polyphenol content was observed from ethyl acetate fraction with 209.21 mg GAE/g, and the lowest total polyphenol content was 27.76 mg GAE/g by *n*-hexane fraction. This result is significantly higher than the one reported by Singh and Chaturvedi [9]. They reported that the total phenolic content of methanolic extract of *R. emodi* rhizome was 92.82 μg GAE/mg. The amount is less than that of ethyl acetate, water, and *n*-butanol fraction of this study. The result implies the optimum yield of polyphenols can be achieved by choosing different extraction solvents.

There are several studies that reported the positive correlations between free radical scavenging activity and antioxidant content of *R. emodi* extracts [26]. According to Lee et al. [27], superoxide dismutase eliminates superoxide (O_2_^−^), which develops from natural substances and is retained in the body. SOD activity is necessary to restrain O_2_^−^ development because O_2_^−^ is an obstacle and enemy in the body. In this study, the antioxidant activity of *R. emodi* was also verified by comparing DPPH and ABTS^+^ radical scavenging activity for the non-enzymatic system, and SOD activity for the enzymatic system. Throughout of different evaluation methods, ethyl acetate fraction showed the lowest IC_50_ value among the fractions, even significantly lower than the control. The result implies ethyl acetate fraction of *R. emodi* efficiently scavenged free radicals. This is similar to the results reported by others, DPPH radical scavenging activity (%) of *R. emodi* rhizome was 94.57 by methanolic extract by Singh and Chaturvedi [9], and 90% and methanolic extract at 100 μg concentration reported by Rajkumar et al. [4]. Furthermore, a significantly negative correlation was observed between total phenolic contents and antioxidant activity screening methods. These results imply that *R. emodi*, particularly those present in ethyl acetate fraction among the other fractions, contained efficient free radical scavenger, have powerful antioxidant activity and the strongest superoxide anion scavenging activity.

The causal antioxidant compounds were identified by HPLC analysis using ethyl acetate fraction of *R. emodi* wall extract. Well-defined natural flavonoids were firstly identified as major component in the extract, myricitrin, myricetin 3-galloyl rhamnoside, and myricetin. Recent studies suggest and support the potential use of myricetin related to the mechanisms of intrinsic resistance to carcinogen, diabetes, and cardiovascular protection, based on its potent iron-chelating capability, antioxidant and free-radical scavenging activities [28,29,30]. Myricitrin, a natural flavonoid glycoside, is known to possess anti-inflammatory, antifibrotic, and antioxidant activity with stronger free radical scavenging activity than other flavonol rhamnosides [31,32]. These compounds are first identified from *Rheum emodi* wall and suggest that the causal antioxidant compounds could be useful in utilizing *R. emodi* in the industry to take advantage of a medicinal herb.

The antioxidant activity of flavonoids is determined based on the availability of phenolic hydrogens and the stabilization of the resulting phenoxyl radicals through hydrogen bonding or expanded electron delocalization. Structures that determine the antioxidant activity of flavonoids include the number, arrangement, and structural conjugation of the hydroxyl group in the phenol ring [33]. Comparing the antioxidant activity of the identified compounds, the structure of flavonoids affected the antioxidant activity (data not shown). According to Miller and Rice-Evans [28], the gallate acylation on the glycoside moiety plays an essential role in enhancing antioxidant activity relative to that of corresponding glycoside. Many flavonols exist as glycosides at the 3-*O*-position in the plant body and such glycosylation lowers the antioxidant activity.

## 4. Materials and Methods

### 4.1. Sample Preparation

*R. emodi* was collected from Langtang, at an altitude of 3500 m, Nepal. The plant was identified with standard references and authenticated by a botanist. The plant materials were air-dried, and 800 g of the sample was powdered and extracted with 70% (*v*/*v*) ethanol under reflux (3 × 2.5 L, 2 h each time), then filtered through Whatman No. 2 filter paper. The solvent of the combined extract was evaporated under reduced pressure using a rotary vacuum-evaporator at 50 °C and the remaining water was removed by freeze-drying. The vacuumed crude extract of EtOH extract was successively extracted with *n*-hexane (room temperature, 2 × 1 L), dichloromethane (2 × 1 L), ethyl acetate (2 × 1 L), *n*-butane (2 × 1 L), and water. The solvent fractions were stored below −18 °C for further analysis.

### 4.2. Total Phenolic Compound Analysis

The total polyphenol content was determined by the Folin–Ciocalteu procedure. Fifty micro liters of sample extracts and Folin–Ciocalteu’s phenol reagent were placed into the 96-well plate. The mixtures were allowed to react at room temperature for 3 min, then 100 μL of 2% Na_2_CO_3_ solution was added. After 30 min, the absorbance was measured using UV-visible spectrophotometer at 750 nm, and the content of the total phenolics was estimated as mg of the gallic acid calibration curve. The quantification of phenolic compounds was carried into triplicate and averaged.

### 4.3. DPPH Radical Scavenging Activity

The effect of solvent fractions on DPPH free radical was determined according to the method of Brand-Williams et al. [34], as described by Yoo et al. [35]. Four milliliters of the fractions were added to 1 mL DPPH, and the mixture was homogenized and left to stand in the dark for 30 min. Absorbance was measured using a spectrophotometer at 520 nm, and ascorbic acid was used as a standard. Three times of technical replicate was performed. The scavenging activity of free radicals by the sample was calculated as the ratio of multiplication of the absorbance of the control and the absorbance of the extract with the absorbance of control times one hundred.

### 4.4. ABTS Radical Scavenging Activity

The total antioxidant activity of the samples was measured using ABTS^+^ radical cation decolorization assay as reported by Re et al. [36]. ABTS was dissolved in water to 7 mM concentration and ABTS radical cations were produced by reacting ABTS stock solution with 2.45 mM potassium persulfate (final concentration). The mixture was let to stand at the dark room temperature for 12–16 h before use. ABTS oxidation was commenced immediately, but the absorbance was not maximal and stable until 6 h lapse. The radical cation was stable in this form for more than 2 days of storage in the dark with room temperature for 16 h to allow the completion of radical generation. It was diluted with ethanol (99.5%) so that its absorbance was adjusted to 0.70 ± 0.02 at 734 nm. To determine the scavenging activity, 190 μL of ABTS reagent was mixed with 10 ul of sample and the absorbance was measured at 734 nm after 30 min of reaction time at room temperature. Ascorbic acid was used as a standard. The experiment was completed with 3 times of replication.

### 4.5. Superoxide Dismutase (SOD) Assay

SOD activity was determined using the SOD kit (Dojindo, Kumamoto, Japan) by following the manufacturer’s instructions. Twenty microliters of each sample solution were added to 20 μL double distilled water. The mixture was placed into each well with 200 μL of WST working solution. Twenty microliters of Enzyme Working Solution were added into each well and mixed thoroughly. The plate was incubated at 37 °C for 20 min. The absorbance was read at 450 nm using a microplate reader, and ascorbic acid was used as a standard. All the experiments were completed in triplicate.

### 4.6. Isolation and Identification of Antioxidant Compounds

The components of ethyl acetate fraction of *R. emodi* were isolated and identified using a Micromass ZQ MS (Waters Co., Milford, MA, USA) and an Alliance e2695 HPLC system (Waters Co., Milford, MA, USA) equipped with a 2998 photodiode array detector (PDA), in addition to a YMC PACK ODS-AM reversed-phase column (4.6 × 250 nm I.D., 5 μm; YMC Co. Ltd., Kyoto, Japan). The analysis was conducted at the flow rate of 1 mL/min at the detection wavelength of 190–600 nm (the representative wavelength of 254, 350 nm) and the oven temperature was 30 °C. The mobile phases used were 0.1% trifluoroacetic acid in water (phase A) and acetonitrile (phase B). The pretreated sample was analyzed by using the following gradient conditions; a gradient of 10% to 30% of phase B over 25-min period; 30% of phase B for five minutes; gradient of 30% to 10% of phase B for three minutes; and then final wash with 10% phase B for seven minutes. MS analysis was run in a positive ionization mode using electrospray ionization (ESI) source. The MS parameters were set to cone voltage of 30 V, source temperature of 120 °C, dissolve temperature of 350 °C, and dissolved N_2_ gas flow of 500 L/h. The range of molecular weight was 100–1200 *m*/*z* in the full scan mode.

In addition, the fractionation parameters were set to maximum fractions and tubes per injection of 78, solvent front delay of 60 s, split/collector delay of 10 s, and maximum fraction width of 60 s. The ethyl acetate fraction from *Rheum emodi* wall with the concentration of 50,000 ppm was used for 500 μL per injection. In two step purification, the fractionation parameters were different with the first step as follows: maximum tubes per injection of 114, maximum fraction width of 30 s. The ethyl acetate fraction from *Rheum emodi* wall with concentration of 50,000 ppm was used for 200 μL per injection. Collected fractions were analyzed directly from collection tubes, without any additional liquid handling. Relative peak areas in mass chromatograms were used to evaluate peak purity of collected fractions relative to the major component.

### 4.7. Statistical Analysis

The data was recorded as mean standard deviations and analyzed by SPSS (version 12.0 for Windows XP, SPSS Inc, Chicago, IL, USA). One- and two-way analysis of variance (ANOVA) and Duncan’s multiple comparisons were carried out to test for any significant differences between the means; the mean values of antioxidant activity between two extracts or two treatments were analyzed by an independent samples *t*-test. Correlations were obtained by Pearson correlation coefficient in bivariate correlations. Differences between means at 5% (*p* < 0.05) level were considered significant. ChemDraw Ultra 8.0 software (PerkinElmer, Waltham, MA, USA) was used for drawing the chemical structures of compounds.

## 5. Conclusions

*Rheum emodi* wall has been used in Asian countries as a medicinal plant. The extracts of *R. emodi* have been considered a promising source of antioxidation based on the previous studies in screening antioxidant and radical scavenging activity. This study accomplished in identifying the efficient extraction solvent of *R. emodi*, which is ethyl acetate. Ethyl acetate extract showed the highest phenolic content and strong radical scavenging activity throughout different methods of measuring antioxidant activity. Moreover, LC/MS results suggested that the major phenolic compounds in the ethyl acetate fraction of *R. emodi* were identified to myricitrin, myricetin 3-galloyl rhamnoside, and myricetin. These compounds are firstly reported to be present in *R. emodi* in this study. Aforementioned compounds are well-defined natural flavonoids, identified and proved with their antioxidant activity and free-radical scavenging activity. Therefore, the associated active ingredients of *R. emodi* were identified aside from few anthraquinones. This finding could contribute to practical use of *R. emodi* as a natural and safe source of antioxidants.

## Figures and Tables

**Table 1:** Total phenolic contents of *R. emodi* fractions using the different solvents.

Fraction	Total Phenolic Content (mg GAE/g ^1^)
*n*-hexane	27.76 ± 0.49 ^a^
*n*-butanol	133.49 ± 0.58 ^c^
Ethyl acetate	209.21 ± 5.81 ^d^
Dichloromethane	66.27 ± 0.34 ^b^
Water	139.00 ± 2.12 ^c^

^1^ Total phenolic content is expressed as milligrams of gallic acid equivalents (GAE) per gram of dry weight for the sample. ^a–d^ Values within a column followed by different letters are significantly different (Duncan test, *n* = 3, *p* < 0.05).

**Table 2:** Radical scavenging and antioxidant activity of *R. emodi* using different solvent fractions.

Antioxidant Activities	Fraction	IC_50_ (μg/mL)
DPPH radical scavenging	*n*-hexane	2448.79 ± 174.14 ^c^
	*n*-butanol	38.67 ± 18.80 ^a^
	Ethyl acetate	21.52 ± 2.78 ^a^
	Dichloromethane	348.42 ± 26.98 ^b^
	Water	43.04 ± 0.39 ^a^
	Ascorbic acid *	70.33 ± 0.30 ^a^
ABTS^+^ radical scavenging	*n*-hexane	1718.05 ± 308.38 ^b^
	*n*-butanol	128.96 ± 1.95 ^a^
	Ethyl acetate	90.25 ± 0.62 ^a^
	Dichloromethane	260.67 ± 6.51 ^a^
	Water	141.97 ± 2.20 ^a^
	Ascorbic acid *	111.06 ± 2.05 ^a^
SOD activity	*n*-hexane	ND
	*n*-butanol	24.36 ± 0.84 ^b^
	Ethyl acetate	2.31 ± 1.1 ^a^
	Dichloromethane	43.87 ± 9.03 ^c^
	Water	32.25 ± 4.11 ^b^
	Ascorbic acid *	64.78 ± 4.65 ^d^

^a–d^ Values within a column followed by different letters are significantly different (Duncan test, *n* = 3, *p* < 0.05). * Compound used as a positive control.

**Table 3:** Correlation between the total polyphenol content and antioxidant activity of *R. emodi* fractions.

	Total Phenolic Content	Antioxidant Activity		
		DPPH	ABTS^+^	SOD
**Total Phenolic** **Content**	1			
**DPPH**	−0.765 **	1		
**ABTS^+^**	−0.740 **	−0.988 **	1	
**SOD**	−0.928 **	−0.700 *	−0.853 **	1

The values represent the correlation coefficient (*r*). * *p* < 0.05 and ** *p* < 0.01, respectively.

## Sample Availability

Samples of the compounds are not available from the authors.
